# On the Assessment of Surface Quality and Productivity Aspects in Precision Hard Turning of AISI 4340 Steel Alloy: Relative Performance of Wiper vs. Conventional Inserts

**DOI:** 10.3390/ma13092036

**Published:** 2020-04-27

**Authors:** Adel T. Abbas, Magdy M. El Rayes, Monis Luqman, Noha Naeim, Hussien Hegab, Ahmed Elkaseer

**Affiliations:** 1 Mechanical Engineering Department, College of Engineering, King Saud University, Riyadh 11421, P.O. Box 800, Saudi Arabia; melrayes@ksu.edu.sa (M.M.E.R.); monisluqman9@gmail.com (M.L.); 2 Department of Production Engineering and Mechanical Design, Port Said University, Port Fuad 42526, Egypt; nfouad39@gmail.com; 3 Mechanical Design and Production Engineering Department, Cairo University, Giza 12613, Egypt; hussien.hegab@uoit.ca; 4 Institute for Automation and Applied Informatics, Karlsruhe Institute of Technology, 76344 Karlsruhe, Germany; ahmed.elkaseer@kit.edu

**Keywords:** precision hard turning, AISI 4340 alloy steel, wiper insert, conventional round nose insert, roughness, productivity

## Abstract

This article reports an experimental assessment of surface quality generated in the precision turning of AISI 4340 steel alloy using conventional round and wiper nose inserts for different cutting conditions. A three-factor (each at 4 levels) full factorial design of experiment was followed for feed rate, cutting speed, and depth of cut, with resulting machined surface quality characterized by resulting average roughness (Ra). The results show that, for the provided range of cutting conditions, lower surface roughness values were obtained using wiper inserts compared with conventional inserts, indicating a superior performance. When including the type of insert as a qualitative factor, ANOVA revealed that the type of insert was most important in determining surface roughness and material removal rate, with feed rate as the second most significant, followed by the interaction of feed rate and type of insert. It was found that using wiper inserts allowed simultaneous increases in feed rate, cutting speed, and depth of cut, while providing better surface quality of lower Ra, compared to the global minimum value that could be achieved using the conventional insert. These findings show that wiper inserts produce better surface quality and a material removal rate up to ten times higher than that obtained with conventional inserts. This clearly indicates the tremendous advantages of high surface quality and productivity that wiper inserts can offer when compared with the conventional round nose type in precision hard turning of AISI 4340 alloy steel.

## 1. Introduction

Precision hard turning concerns the turning of material with superior mechanical properties [1], particularly high levels of strength [2] and hardness [3] such as alloy steels [4] and nickel-based super alloys [5,6], materials with high strength/weight ratio [7] and wear resistance [8] such as titanium alloys [9]. Although these materials fulfil a wide range of industrial requirements [10], they are difficult-to-cut and require advanced manufacturing processing techniques [11]. Previously, besides nonconventional techniques that are expensive and time consuming, grinding was almost the only mechanical method to process difficult-to-cut materials. However, grinding had a low throughput and limited capabilities in terms of flexibility and the geometries to be machined [12]. A cost-effective alternative for materials with high hardness was shown to be precision hard turning, which has the added advantage of a relatively high throughput with a reduction in processing time up to 60%, when compared with grinding [13]. This is due mainly to its ability to precisely machine components to produce complex geometries with tight tolerances and higher surface quality and material removal rate (MRR) [14]. This has led to an increased adoption of precision hard turning by industry for different applications, including automotive, marine, power engine, aerospace, and military components. 

High precision automotive parts can, for example, reduce friction and increase the life of bearings and injection systems, which in turn enhance the overall efficiency of internal combustion engines and decrease emissions. Thus, high precision could be an important factor in sustainability and environmental impact [15]. In addition, applications which can benefit from the high dimensional accuracy capabilities of precision hard turning include telecommunications, microelectronics, displays, laser scanners, lenses, and sensors. Precision hard turning shows great potential for meeting high precision required in the production of tooling systems, in manufacturing operations such as cutting tools, jigs and in the many applications which would benefit from higher surface accuracy and longer life [16].

Steel alloys are widely utilized in a wide range of applications that require components possessing enhanced material properties such as high hardness, high strength, high facture toughness fatigue and wear resistance, and thermal stability to be manufactured to a high precision, [17]. Special attention has been paid to high strength alloy steels such as AISI 4340 due to its advanced mechanical properties which lead to its use in a large number of industries, including; automotive, construction and structural engineering, power generation, defense applications and weaponry [18,19]. However, the machining of steel alloys can be difficult due to their uniquely high level of strength which is known to quickly damage cutting tools and generate high levels of stress when machining due to the elevated temperatures produced [20]. This requires the materials from which the cutting tools are made should possess excellent physical and mechanical properties including high hot hardness, fracture toughness, and abrasion resistance, the latter because steel alloys are considered relatively abrasive [21]. Low ductility of alloy steels is another reason for their low machinability as it dominates the chip formation process, producing a more brittle fracture (associated with more micro cracks and resulting in poor surface quality) rather than a ductile shear deformation mechanism. 

The low machinability of steel alloys combined with a high demand, has meant the precision hard turning of steel alloys has received much attention [22]. This study is part of the drive to develop new processes, methodologies, and tooling to improve the machinability of hardened steel alloys aimed at increasing the material removal rate (MRR), improving surface quality while extending tool life, and minimizing the number of steps in the process chain. Hybrid machining techniques such as laser assisted [23], magnetic field assisted [24], and ultrasonic-assisted machining [25] have been used to machine hardened steels. As have, cryogenic cooling techniques [26], and special tools such as rotary tools (propelled or driven) [27]. It has been reported that wiper inserts, where the cutting edge is a series of radii rather than a single radius, as in conventional inserts, have been successful utilized for precision hard turning operations [28].

**Figure 1:**
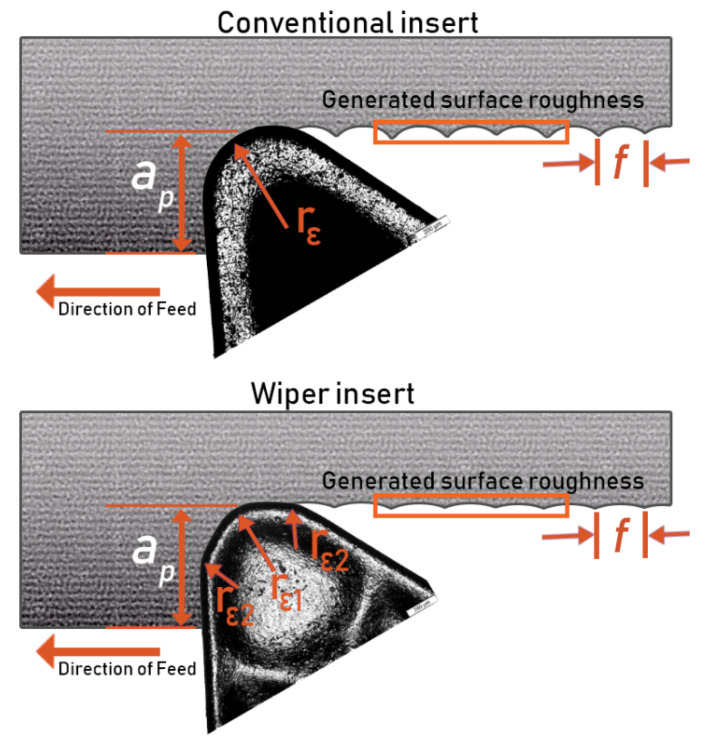
Machining action of (**a**) conventional and (**b**) wiper inserts.

In turning operations, feed rate and nose radius of the cutting tool are the governing parameters that dominate the surface generation process, and thus the obtainable roughness [29,30,31]. It is well known that an insert with a conventional round nose geometry restricts the productivity of the turning process. This is due to the fact that the upper limit of working feed rates is restricted by the nose geometry, and applying a high feed rate will produce poor surface quality [32], see Figure 1a.

One possible solution is the use of a cutting insert with a larger nose radius. This, however, will lead to higher cutting forces and consequently regenerative chatter. This either-or situation, where applying optimal process conditions either for feed rate, or for cutting tool geometry can achieve either, but not both, higher surface quality or higher productivity. Recently, with the arrival of wiper-based geometry tools, this conflict can be reduced. Wiper-based geometry tools have wiper edges that are composed of small radii smoothly connected to the ordinary nose, see Figure 1b. Thus, wiper edges result in a nose with, effectively, a large radius of curvature that enables application of high feed rates for high productivity, and at the same time, leads to the generation of a high-quality surface as a result of optimized nose geometry.

A number of projects have compared the cutting performance of wiper and conventional inserts based on experimental work [12] or finite element simulation [32,33]. Wiper inserts produced a noticeable improvement in achievable surface quality compared with conventional insert [34], revealing the possibility of achieving smoother surfaces at higher feed rates. It was demonstrated that the wiper insert can be a successful alternative to the grinding process to obtain excellent surface finishing [35,36,37]. As with conventional inserts, the parameters most important for determining surface roughness when using wiper inserts, were feed rate and nose radius followed by cutting speed [29,30]. 

Workpiece hardness has been found to have a considerable effect on output when machining using wiper inserts [38]. Others investigations of machining performance have included the effect of wiper insert on metal removal rate, cutting force, temperature and tool wear, and surface integrity [39,40]. Compared with conventional inserts, better surface topography with higher metal removal rates are achieved by wiper inserts [41], but cutting force and power consumed are increased [12]. Furthermore, wiper inserts when compared with conventional inserts were found to have the negative effect of increasing temperature on the tool rake face during machining, which will generate higher residual stresses and increase tool wear [42]. Nevertheless, in some previous studies, a better performance regarding tool wear was found when using wiper inserts than conventional [12,38]. Moreover, using wiper inserts showed high chip curling, which makes the chip separate earlier from the tool rake face [42].

The literature reviewed showed a number of studies have confirmed the advantages of using wiper inserts to obtain better surface quality when compared with conventional tools. However, regarding the precision hard turning of AISI 4340, so far there has been no detailed study conducted to quantitatively examine the performance of wiper inserts compared to conventional inserts in terms of simultaneous surface quality and MRR, under a wide range of cutting conditions. In this context, the aim of this study is to conduct an assessment of the relative merits in terms of surface roughness and MRR of cemented carbide conventional and wiper inserts during precision hard turning of AISI 4340. Thus, this work offers the first attempt in the open literature to experimentally and physically investigate the effectiveness of using wiper insert in precision hard turning of AISI 4340. However, in future work, this will be followed by further studies to examine the effect of a range of workpiece materials with different mechanical and physical properties as well as the influence of different insert grades, materials, coatings, and geometrical attributes on machining matters such as cutting mechanisms, tool wear regimes, and surface integrity in precision hard turning processes.

Following this introduction, the remainder of the paper is organized as follows. First, the experimental work, with description of workpiece shape, material and composition, the test rig, insert designations, and applied cutting conditions and then surface roughness characterizing are given. Second, the experimental results are presented and discussed, this is followed by a description of the statistical study to analyze the data and establish the regression models. ANOVA is used to investigate interaction effects of the process parameters. Finally, conclusions are drawn based on the research findings.

## 2. Experimental Work

The material used in this study is AISI 4340 alloy steel [19,43,44]. The measured hardness was 43.5 HRC and its elemental composition as determined using spectroscope analyzer is shown in Table 1. 

**Figure 2:**
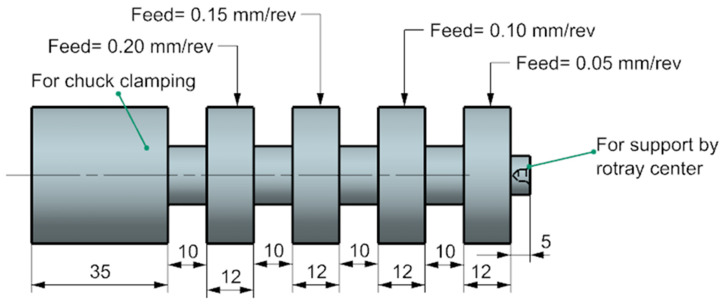
A schematic of the test specimen prepared for turning tests.

For the turning trials, the specimens to be machined were cylinders of 50 mm diameter and 130 mm long. In each workpiece, four machining segments of 12 mm length each were prepared by creating clearance grooves of 10 mm separating consecutive machined segments, as shown in Figure 2.

**Figure 3:**
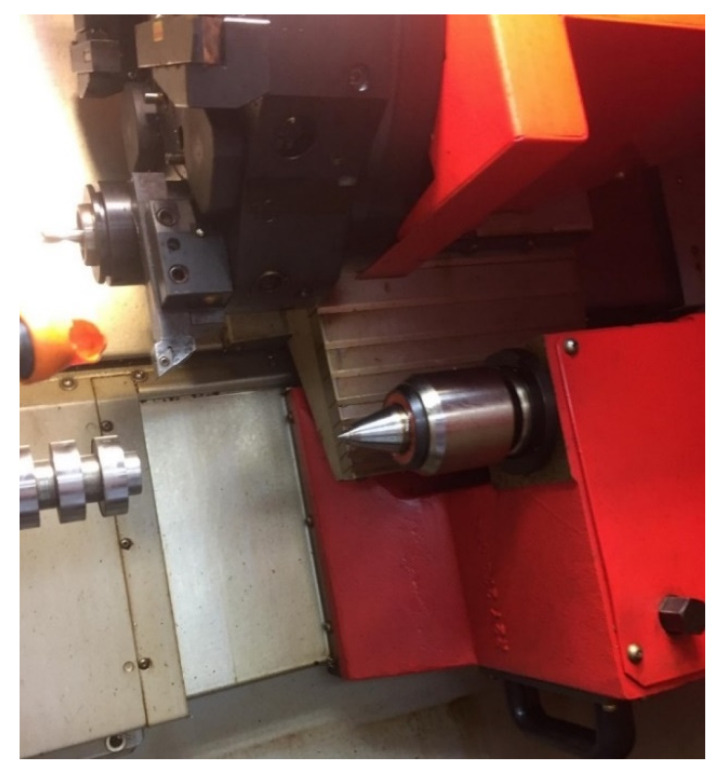
Experimental test rig: an EMCO Concept Turn 45 CNC lathe and clamped workpiece with cutting insert for turning test.

**Figure 4:**
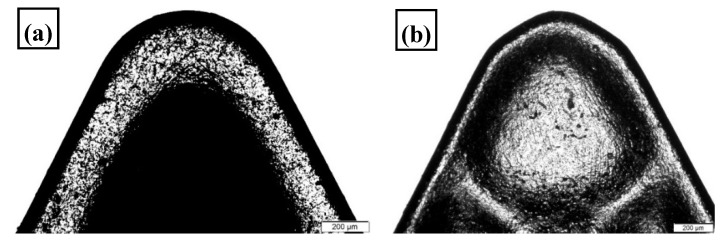
Carbide inserts (**a**) conventional (DCMT 11 T304-PF GC4325) and (**b**) wiper (DCMX 11 T304-WF GC4325).

The machine used for the turning trials was an EMCO Concept Turn 45 CNC lathe (Emco, Salzburg, Austria), Figure 3. The test rig was attached to a SDJCL 2020K11 tool holder that was used for both types of inserts conventional (DCMT 11 T304-PF GC4325, Figure 4a, Sandvik, Stockholm, Sweden) and wiper (DCMX 11 T304-WF GC4325, Figure 4b, Sandvik, Stockholm, Sweden) carbide inserts. Both types of inserts had 0.4 mm nose radius, 55° cutting edge angle, 7° clearance angle, and positive rake angle of (6°). 

The various cutting conditions for the machining tests followed a full factorial experiment with three parameters (feed rate, cutting speed, and depth of cut) and each parameter having four levels, see Table 2. Cutting speed ranged from 75 to 150 m/min in steps of 25 m/min, feed rate from 0.05 to 0.20 mm/rev in steps of 0.05 mm/rev, and depth of cut from 0.10 to 0.25 mm in steps of 0.05 mm. This gave a total 64 trials for each type of insert, so there was a total of 128 turning tests. All turning trials were conducted under a flood coolant condition using ECO-COOL-MK-3 cutting coolant fluid dilute in distilled water (1 Vol. of ECO-COOL-MK-3 to 5 Vol. of distilled water, Saudi Petroleum Company, Jeddah, Saudi Arabia). The mixture was well stirred to ensure complete dispersion of the coolant fluid. 

The surface roughness tester Tesa was used to determine surface roughness (Ra) for an 8 mm cut-of-length.

## 3. Results and Discussion

### 3.1. Generated Surface Roughness of AISI 4340 Steel Alloy: Wiper vs. Conventional Inserts

**Figure 5:**
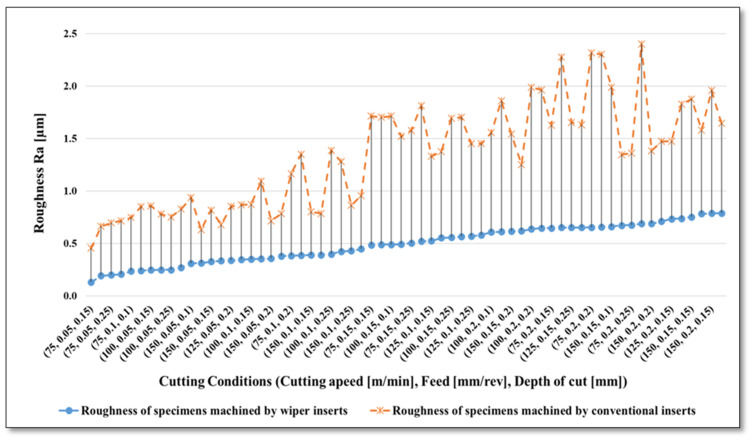
Resultant average roughness (Ra µm) for AISI 4340 specimens machined by wiper and conventional inserts.

**Figure 6:**
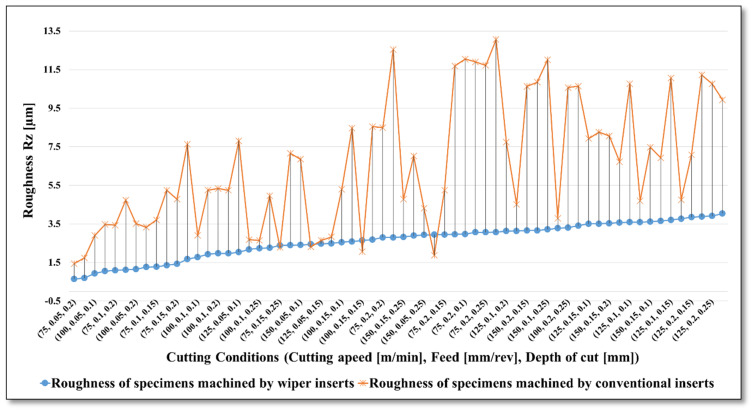
Resultant average roughness (Rz µm) for AISI 4340 specimens machined by wiper and conventional inserts.

The measured surface roughness (Ra and Rz) for all the experiments are presented in Figure 5 and Figure 6, respectively, in ascending order of roughness generated when using wiper inserts. Both graphs show a comparison between the performances of wiper vs. conventional inserts in term of resultant surface roughness (Ra and Rz in µm) of machined AISI 4340 specimens under the whole range of cutting parameters. Please note that all the results are plotted in Figure 5 and Figure 6, however due to restricted space in the X-axis, only 32 designations of applied cutting conditions are shown in the X-axis values. Looking at Figure 5, it is not difficult to see that wiper inserts outperform the conventional, with substantial improvement in the obtained surface quality for the whole range of cutting conditions. The reduction of achievable surface roughness varies between 71.8% (maximum improvement) when turning with *V_c_* = 75 m/min, *f* = 0.1 mm/rev, *a_p_* = 0.1 mm; and 50.1% (minimum improvement) with *V_c_* = 150 m/min, *f* = 0.15 mm/rev, a*_p_* = 0.1 mm. From the experimental results, in terms of generated mean roughness depth (Rz) presented in Figure 6, relative responses of both types of insert are quite similar to those illustrated in Figure 5, with general better performance of the wiper when compared with the conventional inserts. In particular, lower Rz was obtained when wiper inserts were used for the whole range of cutting conditions (with an average reduction in obtainable Rz of 51.9% relative to the surface achieved with conventional inserts) with minor exemptions in 4 points out of the 64 experimental trials, where the conventional inserts show relatively better results than the wiper ones. 

**Figure 7:**
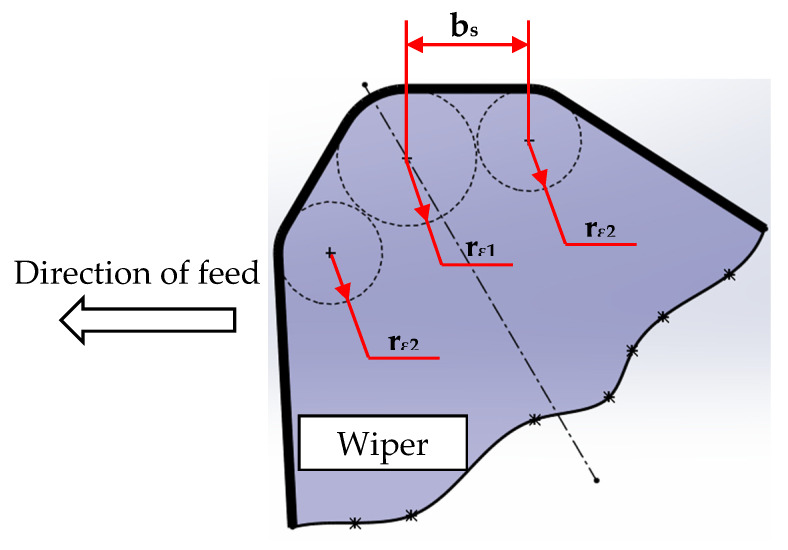
Geometry of wiper insert.

To elaborate the unrivalled performance of wiper inserts when compared with conventional round nose ones, the geometry of a wiper insert is illustrated in Figure 7. It is obvious to see that unlike the geometry of a conventional insert that is composed of a single round nose, wiper geometry consists of small radii (rε2) smoothly connected to the ordinary nose (central circle with radius rε1), see Figure 6. Thus, the wiper edge geometry results in a nose with an effective straight section of minor cutting edge (bs). Therefore, with the aid of the straight section of the minor cutting edge, when applying relatively small feed rates, surface generated contains blunt peaks with substantially smaller profile slopes, resulting in better surface quality [45]. Nevertheless, when the applied feed rate is less than the straight segment of the minor cutting edge, theoretically, almost flat surface profile without peaks can be obtained [46]. This can explain why wiper inserts can be utilized to produce high quality machined surface with stunningly low surface roughness which makes precision hard turning prime candidate for machining applications where high surface quality and productivity are required.

**Figure 8:**
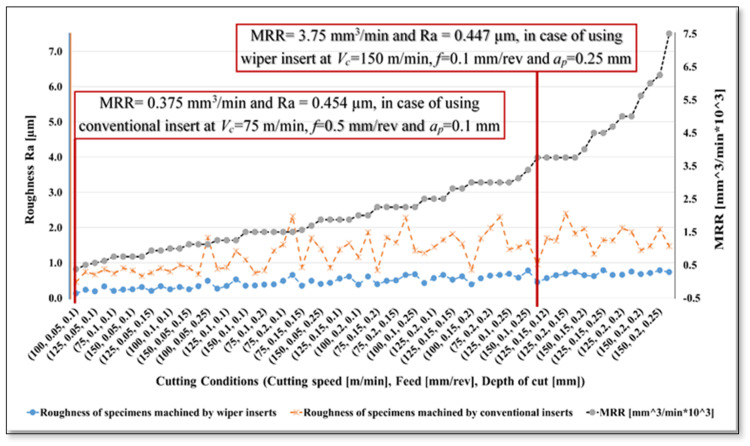
Material removal rate (MRR) and average resultant roughness (Ra µm) of AISI 4340 specimens machined by wiper and conventional inserts.

In Figure 8, the measured surface roughness values are again presented but with the addition of MRR values obtained at each cutting condition. The data presented in Figure 8 are plotted in ascending order of MRR, for the whole range of cutting conditions (MRR values for both types of insert are the same, since MRR depends only on the parameters, *V_c_*, *f*, and *a_p_*). As in Figure 5 and Figure 6, the complete set of the results are plotted in Figure 8, but due to restricted space along the X-axis, only half the designation of applied cutting conditions are seen in the X-axis values. Looking at Figure 8, one can observe that the difference between the maximum and minimum surface roughness (Ra) obtained when turning AISI 4340 using wiper inserts is relatively small (0.788 to 0.129 µm) for the whole range of applied cutting conditions. This means that an increased cutting rate can be achieved without seriously decreasing surface quality, which in turn leads to a corresponding increase in the MRR without deterioration in resultant surface roughness. In particular, using wiper inserts enables the application of higher values for the cutting parameters: *V_c_* up to 150 m/min, *f* up to 0.1 mm/rev, and *a_p_* up to 0.25 mm, which produces a very high value of MRR (3.75 mm 3/min), while the roughness increased only slightly to Ra = 0.447 µm. This roughness value is less than the global minimum achieved using the conventional insert, Ra 0.454 µm at *V_c_* = 75 m/min, *f* = 0.05 mm/rev, and *a_p_* = 0.1 mm, while the corresponding MRR is ten times that obtained with the conventional insert, MRR = 0.375 mm^3^/min.

### 3.2. Statistical Analysis

To better understand the performance of the two insert types when turning AISI 4340 specimens under different cutting parameters, statistical regression models were developed using MATLAB. A quadratic regression method was utilized to fit a second order polynomial, see Equation (1), to the experimental results:

(1) $$y=b_{0}+\sum b_{i}x_{i}+\sum b_{ii}x_{ii}^{2}+\sum b_{ij}x_{i}x_{j},$$

where ‘y’ is the output response (Ra in µm), while *‘b_0_’*,*’b_i_’*, ‘*b_ii_*’, and ‘*b_ij_’* are the regression coefficients or predictors, and ‘*x_i_*’ and ‘*x_j_*’ is the value of the corresponding i^th^ and j^th^ factors, respectively. 

The developed model resulting from the quadratic regression model with a robust fit presents 10 terms when using the three process parameters studied in this work (feed rate, cutting speed, and depth of cut). The values obtained for each coefficient are presented in Equations (2) and (3), where ‘*Vc_n_*’ is the normalized value of cutting speed, ‘*f_n_’* is the normalized value of feed rate, ‘*ap_n_’* is the normalized value of depth of cut, where actual levels of the input parameters were normalized to a [−1, 1] range. ‘*Ra_wiper_*’ and ‘*Ra_conventional_*’ are the predicted average roughness in µm for the AISI 4340 specimens machined by wiper and conventional inserts, respectively. For the samples machined by wiper inserts, R-squared was 0.914, and adjusted R-squared was 0.900 for the regression model developed. For the samples machined by conventional inserts the corresponding values were: R-squared = 0.936, and adjusted R-squared = 0.925:

(2) $$\begin{array}{ccc} Ra_{wiper} & = & 0.518+0.21f_{n}+0.076Vc_{n}+0.032ap_{n}-0.017f_{n}Vc_{n}-0.007f_{n}ap_{n}\\ & & -0.008Vc_{n}ap_{n}-0.011f_{n}^{2}-0.029Vc_{n}^{2}-0.005ap_{n}^{2}, \end{array}$$

(3) $$\begin{array}{ccc} Ra_{\mathrm{conventional}} & = & 1.431+0.589f_{n}-0.147Vc_{n}+0.085ap_{n}-0.215f_{n}Vc_{n}-0.024f_{n}ap_{n}\\ & & -0.039Vc_{n}ap_{n}-0.026f_{n}^{2}-0.127Vc_{n}^{2}-0.017ap_{n}^{2}. \end{array}$$

The developed regression models were then used to analyze and plot the effects of the different parameters and their interactions on the obtainable surface roughness, as described in the following sections. 

**Figure 9:**
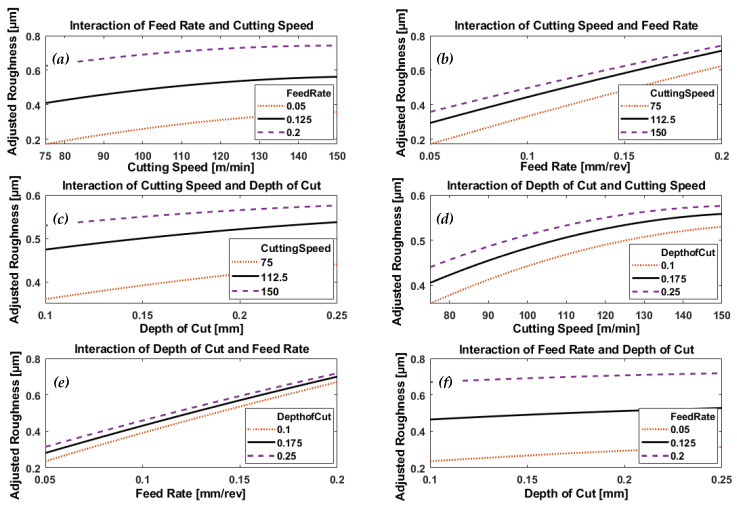
Plots depicting the interactive effects of feed rate, cutting speed, and depth of cut on generated surface roughness of AISI 4340 specimens machined by wiper inserts; (**a**) the effect of cutting speed at different levels of feed rates, (**b**) the effect of feed rate under different levels of cutting speeds, (**c**) the effect of depth of cut at different levels of cutting speeds, (**d**) the effect of cutting speed under different levels of depth of cuts, (**e**) the effect of feed arte at different levels of depth of cuts and (**f**) the effect of depth of cut under different feed rates.

**Figure 10:**
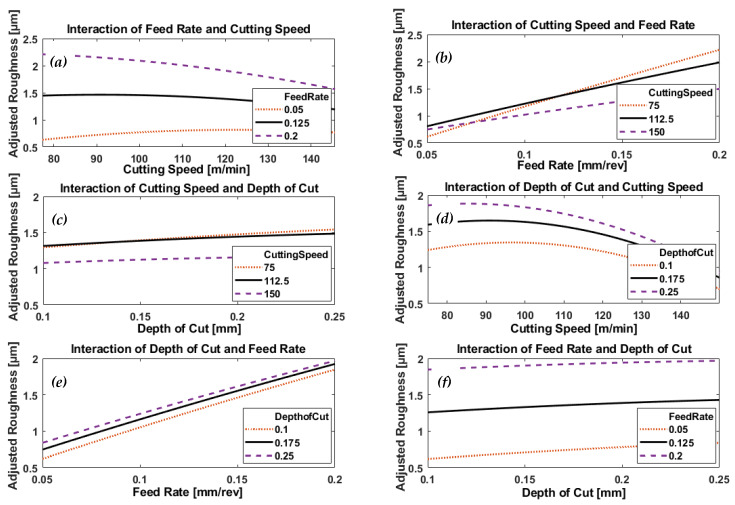
Plots depicting the interactive effects of feed rate, cutting speed, and depth of cut on generated surface roughness of AISI 4340 specimens machined by conventional inserts; (**a**) the effect of cutting speed at different levels of feed rates, (**b**) the effect of feed rate under different levels of cutting speeds, (**c**) the effect of depth of cut at different levels of cutting speeds, (**d**) the effect of cutting speed under different levels of depth of cuts, (**e**) the effect of feed arte at different levels of depth of cuts and (**f**) the effect of depth of cut under different feed rates.

Figure 9 and Figure 10, respectively, depict the effect of interaction of process parameters (feed rate, cutting speed, and depth of cut) on generated surface roughness (predicted by the model) when machining AISI 4340 specimens using wiper and conventional inserts. 

In case of wiper inserts, surface roughness increases with the increase of cutting speed at all levels of feed rate, Figure 9a, while the large variation of the generated roughness at different levels of feed rate shows it to be the dominant effect generating roughness. Figure 10a depicts a different trend, where the increase in cutting speed leads to a reduction in the obtainable roughness, particularly at the high feed rate of 0.2 mm/rev; with an inverse trend in the relationship at low feed of 0.05, where the roughness increases with the increase in the applied cutting speed. It is clear that feed rate is more significant at low cutting speed and less, but still effective, at high speed as shown in the variation of generated roughness at different feed rates. 

Figure 9b and Figure 10b show a similar linearly proportional increase in roughness with feed rate, but the gradient of the line depends on the cutting speed. Looking at the variation of the response at different levels of cutting speed, one can say cutting speed is effective but not so much as feed rate. 

There also appears to be an approximately linear relationship between the depth of cut and generated surface roughness, at different levels of cutting speed, seen in Figure 9c and Figure 10c for wiper and conventional inserts, respectively. From the plots in Figure 9c, we see that cutting speed has a greater effect than depth of cut on obtainable roughness in the case of wiper inserts, but is less significant when conventional inserts are used, as shown in Figure 10c. In addition, Figure 9c shows that surface roughness increases with increase in cutting speed, which appears to be the opposite effect to that seen in Figure 10c.

Figure 9d shows that resultant surface roughness increases monotonically with cutting speed, over the range of speeds investigated, for a given depth of cut using wiper inserts. In Figure 10d, using conventional inserts above a certain cutting speed that depended on the depth of cut, there was an inverse relationship between roughness and cutting speed. However, in both Figure 9d and Figure 10d, the plotted responses showed depth of cut had a noticeable effect on the process performance. 

Figure 9e and Figure 10e showed identical trends; surface roughness increased linearly with feed rate, with depth of cut having no significant effect on the process for either wiper or conventional inserts. 

Figure 9f and Figure 10f show similar small but significant linear increases in generated surface roughness, with applied depth of cut at different feed rates when using both wiper and conventional inserts. However, the gradients of the plotted trends reveal that surface roughness does not depend on any substantial effect on the depth of cut. Instead, the large variation in the plotted responses to the different feed rates clearly shows its greater influence on surface roughness. 

ANOVA analysis of the experimental results showed that feed rate was the most significant parameter on generated surface roughness (*p*-value = 8.996 × 10^−29^ in the case of wiper inserts, and *p*-value = 4.351 × 10^−32^ for conventional inserts). This was followed by cutting speed (*p*-value = 8.190 × 10^−11^ when using wiper inserts and *p*-value = 3.133 × 10^−8^ in the case of conventional inserts). Depth of cut came last with *p*-value = 0.001520 in the case of wiper inserts, but the interaction of feed rate and cutting speed came third with *p*-value = 3.905 × 10^−9^ in the case of conventional inserts.

Then, and in order to quantify the effect of the insert type as a qualitative factor among the input parameters, another more comprehensive regression model and ANOVA analysis were developed based on the experimental results for the whole 128 trials. Now there were four levels for three parameters (feed rate, cutting speed, and depth of cut) and two levels for the insert type (wiper and conventional). Again, and similar to the development of Equations (2) and (3) as explained in Section 3.2, a quadratic regression method was utilized to fit a second order polynomial to the experimental results of the 128 trials, Equation (4). The developed model resulting from the quadratic regression model with a robust fit presents 14 terms for the four process parameters that were examined: feed rate, cutting speed, and depth of cut, and type of insert, and the values obtained for each coefficient are presented in Equation (4):

(4) $$\begin{array}{ccc} Ra & = & 0.974+0.399f_{n}-0.035Vc_{n}+0.058ap_{n}+0.422In-0.116f_{n}Vc_{n}\\ & & -0.016f_{n}ap_{n}+0.19f_{n}In-0.024Vc_{n}ap_{n}-0.112Vc_{n}In\\ & & +0.027ap_{n}In-0.019f_{n}^{2}-0.078Vc_{n}^{2}-0.011ap_{n}^{2}, \end{array}$$

where ‘*f_n_’* is the normalized value of feed rate, ‘*Vc_n_*’ is the normalized value of cutting speed, ‘*ap_n_*’ is the normalized value of depth of cut, ‘*I_n_*’ is the type of inserts used (wiper or conventional), and ‘*Ra*’ is the predicted roughness average in µm for the AISI 4340 specimens. 

R-squared was 0.960, and adjusted R-squared was 0.955 for the regression model. It is worth emphasising that while feed rate, cutting speed, and depth of cut can vary for any value between the normalized maximum and minimum limits (−1 and 1), insert type can be given only binary values “−1” or “1” for the wiper and conventional inserts, respectively. 

**Figure 11:**
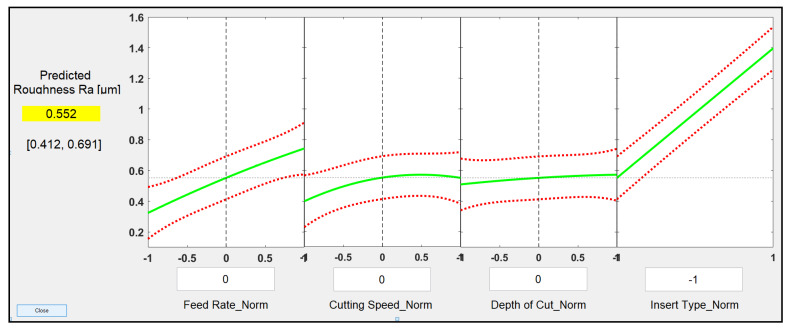
Prediction slice plots created using the developed process regression model for roughness of AISI 4340 specimens machined by both types of inserts, wiper and conventional.

The developed regression model (Equation (4)) was then utilized to create prediction slice plots as shown in Figure 11, which also can be used to show the main effect of each individual process parameter on the generated surface roughness when other parameters are kept constant. The green lines in each plot shows the change in the response value at different levels of the process parameter (normalized value) at constant values of other parameters. The red dashed lines are the 95% confidence limits for the value of the simulated response. The predicted response is obtained by moving the vertical dashed line along the normalized range for each factor to produce the predicted response. It can be used to obtain process parameters at minimum or maximum values of surface roughness. In addition, it is possible to find the predictive results of surface roughness for any cutting condition. Looking at the effect of each individual process parameter, it is not so difficult to see that the insert type is the most significant factor, followed by the feed rate, while cutting speed was found to be third in order, with depth of cut the least significant process parameter. 

This results are in agreement with the ANOVA analysis showing that the type of insert is the most significant parameter affecting surface roughness with a *p*-value of 5.768 × 10^−69^, followed by the feed rate (*p*-value = 3.252 × 10^−53^). The interaction of the feed rate and insert type was found to be the third most effective factor with *p*-value = 3.967 × 10^−25^. These results, clearly indicate the significance of the type of insert, and using a wiper insert considerably improve the generated surface roughness while maintaining a high MRR and thus productivity. 

The developed regression model was then experimentally validated for a set of turning trials conducted under new cutting conditions that were not previously applied, see Table 3. The generated surface roughness of the machined AISI 4340 specimens was measured and compared with the predicted by value obtained from the regression model. It is worth stating that although each validation test was conducted once, it was performed using a fresh insert each time to machine a workpiece of 100 mm long. Then, the generated surface was characterized for the entire machined length, over which different spot measurements were taken along lines parallel to the center line of the workpiece and the arithmetic average was calculated. The results show that the model predictions agreed well with the experimental measured values with an average difference of 7.17% for the AISI 4340 specimens machined by wiper inserts and 10.09% when conventional inserts were used. 

Based on the aforementioned discussion, one can argue that this comparative assessment of the machinability of AISI 4340 alloy steel in terms of obtainable surface roughness using wiper and conventional round nose carbide inserts contributes to a better understanding of the process and thus to the advancement of precision hard turning of high-strength alloy steels. Especially, precision hard turning has shown potential as a profitable and dependable alternative to grinding, since it enormously improves the quality of the product while simultaneously reducing lead times and manufacturing costs. This makes precision hard turning of growing importance for manufacturers, particularly for numerous industrial applications, including automotive, structural engineering, power generation, bearings, high quality driver shafts, defense applications and weaponry. However, appropriate cutting conditions have to be rigorously applied to enable precision turning to deliver very fine machined surfaces. In this context, the results of this research study help identify optimal cutting conditions of feed rate, cutting speed, and depth of cut when using wiper inserts that lead to a significant increase in obtained material removal rate while maintaining a low resultant surface roughness which, in turn, results in simultaneously optimizing productivity and generated surface quality. Thus, this work can be considered as a valuable extension to some previous works [47,48] which focused on modeling and optimizing of the machining processes in terms of achieving higher productivity with maintaining an acceptable performance level. In addition, the findings of this research work assist in obtaining precise, optimal, and cost-effective machining solutions, which can deliver a high-throughput alternative to conventional grinding when processing difficult-to-cut high-strength AISI 4340 alloy steel in a predictable and controllable manner.

## 4. Conclusions

This paper has presented the results of an experimental-based investigation into the effect of cutting conditions (feed rate, cutting speed, and depth of cut) on generated surface roughness when hard turning AISI 4340 alloy steel using wiper and conventional round nose carbide inserts. Importantly, the tests were followed by statistical analysis using ANOVA to produce a model for predicting surface roughness for feed rate, cutting speed, and depth of cut. 

A noticeably better surface quality was obtained from wiper than conventional inserts under the same cutting conditions for the entire range of parameters examined. It was found that, increasing the rate of cutting, in the case of wiper inserts, slightly increased the obtained surface roughness, while a noticeable increase in MRR could be obtained without any deterioration of the generated surface. Specifically, increasing the cutting parameters to feed rate *f* = 0.1 mm/rev, cutting speed, *V_c_* = 150 m/min, and depth of cut *a_p_* = 0.25 mm, when using wiper inserts, produced an average surface roughness value (Ra 0.447 µm) which was less than the minimum roughness that could be achieved using a conventional round nose insert (Ra 0.454 µm). 

In addition to a lower surface roughness, use of a wiper insert under the given values of *f*, *V_c_*, and *a_p_*, produced MRR of 3.75 mm^3^/min which was ten times higher than the MRR obtained using a conventional insert operating under cutting conditions which produced its minimum roughness (*f* = 0.05 mm/rev, *V_c_* = 75 m/min, and *a_p_* = 0.1 mm). 

ANOVA analysis clearly confirmed that the type of insert is the most significant parameter affecting the generated surface roughness, the second was the feed rate, followed by the interaction of the feed rate and insert type. Using wiper inserts can considerably improve the generated surface roughness while increasing MRR and thus productivity. 

Regression analysis was used to develop a relatively simple algebraic model to predict likely surface roughness when using either wiper or conventional inserts for any set of feed rate, cutting speed, and depth of cut. The model was tested and results were promising (average error % between measured and predictions are 7.17% and 10.09% for wiper and conventional inserts, respectively) as a means to pre-select cutting parameters for minimum achievable surface roughness before commencing the machining process. 

## Figures and Tables

**Table 1:** Chemical composition of AISI 4340.

Element	Ni	Cr	Mn	Mo	C	Si	V	Fe
%	2.5	0.9	0.50	0.41	0.40	0.12	0.09	Balance

**Table 2:** Cutting conditions.

Factor	Condition	Unit
Workpiece material	AISI 4340 alloy steel	–
Inserts	Wiper and conventional	–
Cooling	Flood	–
Cutting speed (*V_c_*)	75, 100, 125, 150	[m/min]
Feed rate (*f*)	0.05, 0.10, 0.15, 0.20	[mm/rev]
Depth of cut (*a_p_*)	0.10, 0.15, 0.20, 0.25	[mm]

**Table 3:** Turning validation results of the proposed regression model: measured vs. predicted Ra.

	Feed Rate mm/rev	Cutting Speed m/min	Depth of Cut mm	Surface Roughness, Ra µm					
				Wiper Insert			Conventional Insert		
				Measured	Predicted	Error %	Measured	Predicted	Error %
1	0.065	100	0.25	0.347	0.348	−0.29	1.076	1.017	5.46
2	0.075	100	0.16	0.297	0.333	−12.16	0.935	0.989	−5.75
3	0.060	125	0.15	0.406	0.393	3.32	1.002	0.816	18.56
4	0.120	130	0.12	0.565	0.538	4.78	1.424	1.214	14.77
5	0.085	150	0.24	0.434	0.501	−15.32	0.912	0.966	−5.91
Average of absolute error %						7.17			10.09
